# Skills and compensation strategies in adult ADHD – A qualitative study

**DOI:** 10.1371/journal.pone.0184964

**Published:** 2017-09-27

**Authors:** Carlos Canela, Anna Buadze, Anish Dube, Dominique Eich, Michael Liebrenz

**Affiliations:** 1 Department of Forensic Psychiatry, Institute of Forensic Medicine, University of Bern, Bern, Switzerland; 2 Division of ADHD Research, Psychiatric University Hospital Zurich, Zurich, Switzerland; 3 Santa Barbara Child & Family Services, Santa Barbara, County of Santa Barbara, California; University of California, San Francisco, UNITED STATES

## Abstract

**Objective:**

The primary objectives of this study were to investigate how adult patients with ADHD coped with their symptoms prior to diagnosis and treatment, what skills and compensation strategies they had developed and what their self-perceptions of these strategies were.

**Methods:**

We used a qualitative approach to analyze interviews with 32 outpatients of a specialty care unit at a university hospital.

**Results:**

Patients reported frequent use of diverse compensatory strategies with varying degrees of effectiveness. These were classified into five categories (organizational, motoric, attentional, social, psychopharmacological). In certain circumstances, ADHD symptoms were even perceived as useful.

**Conclusion:**

Before diagnosis and treatment, patients with ADHD may develop a variety of skills to cope with their symptoms. Several of these skills are perceived as helpful. Knowledge of self-generated coping strategies may help better understand patients and their histories and thus facilitate patient cooperation. Moreover, knowing ways in which such patients cope with their symptoms may help elucidate reasons for late or under-diagnosing of the disorder.

## Introduction

Attention-Deficit/Hyperactivity Disorder (ADHD) was once thought to be a transient childhood disorder [1]. During the last few decades it has been established that childhood ADHD has a high rate of persistence into adulthood [2–5], with an estimated prevalence of 2–4.4% in adult populations [6–8]. Adoption, twin and family studies suggest that the risk for developing this disorder is influenced by environmental–especially prenatal risk factors [9, 10]–and genetic factors [11–13]. Increasing data from neurocognitive, neurophysiological and neuroimaging studies support the view that brain dysfunction stands at the core of the syndrome [14–18].

The core symptoms of ADHD often manifest differently in adults than in children [19]. Hyperactivity may be expressed as fidgeting, inability to relax, as restlessness or being unable to sit still for longer periods while studying, at work or in a movie [19, 20]. Impatience, interrupting others in conversations, impulsive behaviour, changing jobs frequently, starting a business on impulse, changing sexual relationships are often expressions of impulsivity, whereas inattention often shows itself as forgetfulness, disorganization, not listening to others in conversations or being late [19, 20].

The cognitive deficits at the core of the disorder may mean that patients have limited resources for coping with daily situations [21], leading to deficits in different social roles. Adult ADHD has been shown to be associated with impaired occupational [22–24], family and relational functioning [25, 26], impaired social skills [27–29], increased driving accidents and poor driving abilities [30, 31]. ADHD has also been associated with an increased risk of co-morbid substance use [32, 33] and other psychiatric disorders [34, 35].

Stimulant medications are used as first-line agents in the treatment of adult ADHD [36] and have been shown to be effective in numerous studies [36, 37]. For non-responders, those with high baseline symptom severity or those that refuse medication, psychotherapy may be an effective alternative or adjunct [38, 39]. Cognitive-behavioural treatments in particular, have proven effective [40–42]. Many psychotherapeutic programs focus on teaching and utilizing a diverse set of coping strategies [43].

It is recognized that many adults with ADHD are not diagnosed as children and often come into contact with psychiatric services only because of co-morbid disorders. Asherson et al. discussed several reasons for under-diagnosing of ADHD [44], among them cultural views of ADHD, lack of knowledge about the disorder or difficulties in the self-reporting of symptoms by ADHD patients since the perception of their own impairments and symptoms seems to differ from that of the environment [45]. Another explanation suggested by Asherson et al. was the possibility that the disorder might not be recognized in high-functioning adults with adequate coping strategies and good psychosocial functioning [44].

Data from psychotherapy research supports the notion that teaching coping strategies and skills may improve psychosocial functioning in individuals with ADHD. Though patients may present with heterogenous levels of impairment–possibly because of the successful use of compensation strategies resulting in adaptive behavior [44]–there has been to the authors‘ knowledge little research about the coping strategies adults with undiagnosed or unmedicated ADHD come up with on their own.

Young et al. found a correlation between cognitive ability and coping in patients with ADHD compared to healthy controls, where personality factors and prosocial behaviour were also associated with coping [21]. It has also been discussed, whether the increased incidence of substance use in individuals with ADHD–for example, nicotine use–might be seen as self-medication [32, 46, 47]. Using a qualitative approach Liebrenz et al. found that nicotine use was perceived as a way to attenuate symptoms by a majority of the interviewed adults with ADHD [48]. Similarly, Nehlin et al. [49] found that one of the desired effects of substance use was attenuation of ADHD symptoms. To the authors’ knowledge, other coping strategies developed by patients with ADHD have not yet been investigated.

The development of coping behaviour and the identification of maladaptive coping is central to cognitive-behavioural treatments [21]. Knowledge about the strategies adults with untreated ADHD generate and use might quicken the therapeutic process and help generate better psychotherapeutic treatments. From a therapist’s point of view, understanding which ‘skills’ are generated prior to diagnosis and seen as helpful would help improve treatment alliance and collaboration. Knowledge about such skills might also further the understanding of this disorder and be helpful in screening high-functioning undiagnosed patients who may not exhibit expected behaviour patterns.

In this qualitative study, we investigated the skills and coping strategies adults with ADHD reported using before having been diagnosed or treated.

## Methods

### Study design

For this study we used an exploratory qualitative approach and conducted a series of semi-structured interviews with adult patients with ADHD that had previously been referred to Zürich’s psychiatric university hospital for diagnosis and treatment. Participants were recruited following purposeful sampling procedures among outpatients being treated in a specialized unit for ADHD diagnosis and treatment at the university hospital. The cantonal ethics committee of Zürich approved the study (Application Nr. E-04/2005).

### Participants

We included subjects aged at least 18 years with a diagnosis of ADHD according to the 10th revision of the International Classification of Diseases [50] and with sufficient fluency in German. Treating psychiatrists identified potential subjects. 184 subjects were then contacted by letter. Of these 49 were reached by telephone by a member of the research group; 17 agreed to participate, 20 claimed a lack of interest in the study and 12 cited a lack of time. 15 participants were referred directly to the research group by their treating physicians. The sample was selected to provide diversity in gender, age, duration of treatment, comorbidity and occupational status. In total, 32 subjects provided their written informed consent to the digital recordings and completed the interview. Table 1 shows their demographic characteristics. Their treating psychiatrists provided the complete charts and medical history.

**Table 1:** Demographic characteristics.

Sex			
	Female	14	43.75%
	Male	18	56.25%
Age			
	25 years old or less	3	9.38%
	26–35 years old	9	28.13%
	36–45 years old	9	28.13%
	46 years old or more	11	34.38%
Age at diagnosis			
	Childhood (<16 years old)	4	12.5%
	16–25 years old	9	28.13%
	26–35 years old	4	12.5%
	36–45 years old	9	28.13%
	46 years old or more	6	18.75%
Employment			
	Unemployed	3	9.38%
	Fully employed	7	21.88%
	Part-time employed	7	21.88%
	School/University	5	15.63%
	No information	10	31.25%

An appointment for the interview was made by telephone by a member of the research group. All interviews were conducted outside the treatment setting by a member of the research group not involved in treatment and unknown to the participant to avoid a response bias. The participants were told, that both interviewers had experience in ADHD treatment and diagnostics, that AB worked in the hospital’s Division of ADHD Research and that AB and CC were interested in ascertaining how ADHD patients coped with their disorder and their opinions about several related topics. The interviews were not made available to the treating psychiatrists. Subjects did not receive any compensation for their participation.

### Interview

A semistructured, flexible, self-developed interview was used to explore the coping strategies participants reported using prior to diagnosis and treatment as adults. The use of a semistructured interview is common in qualitative research and allows the interviewer to adapt to the answers of the interviewee and to further explore interesting themes [51–53]. Sample questions included: What problems did you have? What helped you most? How did you manage before treatment? The research team met regularly during the interview period to discuss the collected data and the focus of the subsequent interviews. This enabled the interviewers to explore themes identified in earlier interviews and to adapt the topic guide accordingly, combining the principles of maximum variation and complexity reduction.

Interviews were conducted in Swiss German, a dialect of Alemannic origin spoken in the German speaking parts of Switzerland. The interviews were conducted on a one-on-one basis by AB (female) and CC (male), both psychiatrists (medical degree) with experience in ADHD treatment and qualitative research working at the time in Zürich’s psychiatric university hospital. Meeting outside the regular treatment setting in offices of outpatients clinics allowed for an atmosphere in which participants could speak freely.

Interviews were started with open-ended, broad, general questions intended to encourage the interviewee to talk freely about his views and experiences [51]. Non-judgmental and non-leading probes helped investigate interview topics without promoting response bias. Paraphrasing and summarizing main points during the interviews helped minimize misunderstandings and clarify ambiguous statements. Field notes were taken after the interview, the length of which varied between 45 and 90 minutes.

### Data analysis

Interviews were digitally recorded using Olympus DS-7000 and then transcribed verbatim into Standard German. Whereas Swiss German is commonly only spoken, Standard German is traditionally used in writing and transcription, which is why all interviews were automatically written down in Standard German. Coding and analysis of data was done manually using a word processor (Microsoft Word for Windows 2013). After removing identifying information, all transcripts were assigned a code number. The transcripts were not returned to the participant. There were also no repeat interviews.

Coding was done according to Mayring’s qualitative content analysis [54], which enables researchers to approach the data without assumptions. AB, CC and ML analyzed the interviews separately and blindly. The interviews were first read, to familiarize the researchers with the data. An inductive approach while deducing different categories allowed the data to ‘speak for itself.’ In regular meetings during the period of interviews, the obtained categories were discussed by the research group and redefined where appropriate to diminish the risk of bias. Consistent with recommendations for qualitative research, it was decided to further explore the emerging topic of self-developed strategies. From the gathered data, two categories (organizational, psychopharmacological) were identified early, with the other categories emerging later in the process and requiring further discussion. Codes were then compared and discussed with ML applying the final code.

Recruitment was ceased after saturation had been reached. Saturation is commonly defined as the point when no new themes can be found.

Appropriate quotes were selected for the manuscript to validate the research findings and translated into English by CC. Back-translation by AB and ML and consequent discussion in the research group ensured accurate translations. ML chose the nearest translation when consensus could not be achieved. Where necessary, quotes were grammatically corrected to improve readability. Quotations were then proof read by a native English speaker.

This study was reported according COREQ guidelines [55].

## Results

**Fig 1:**
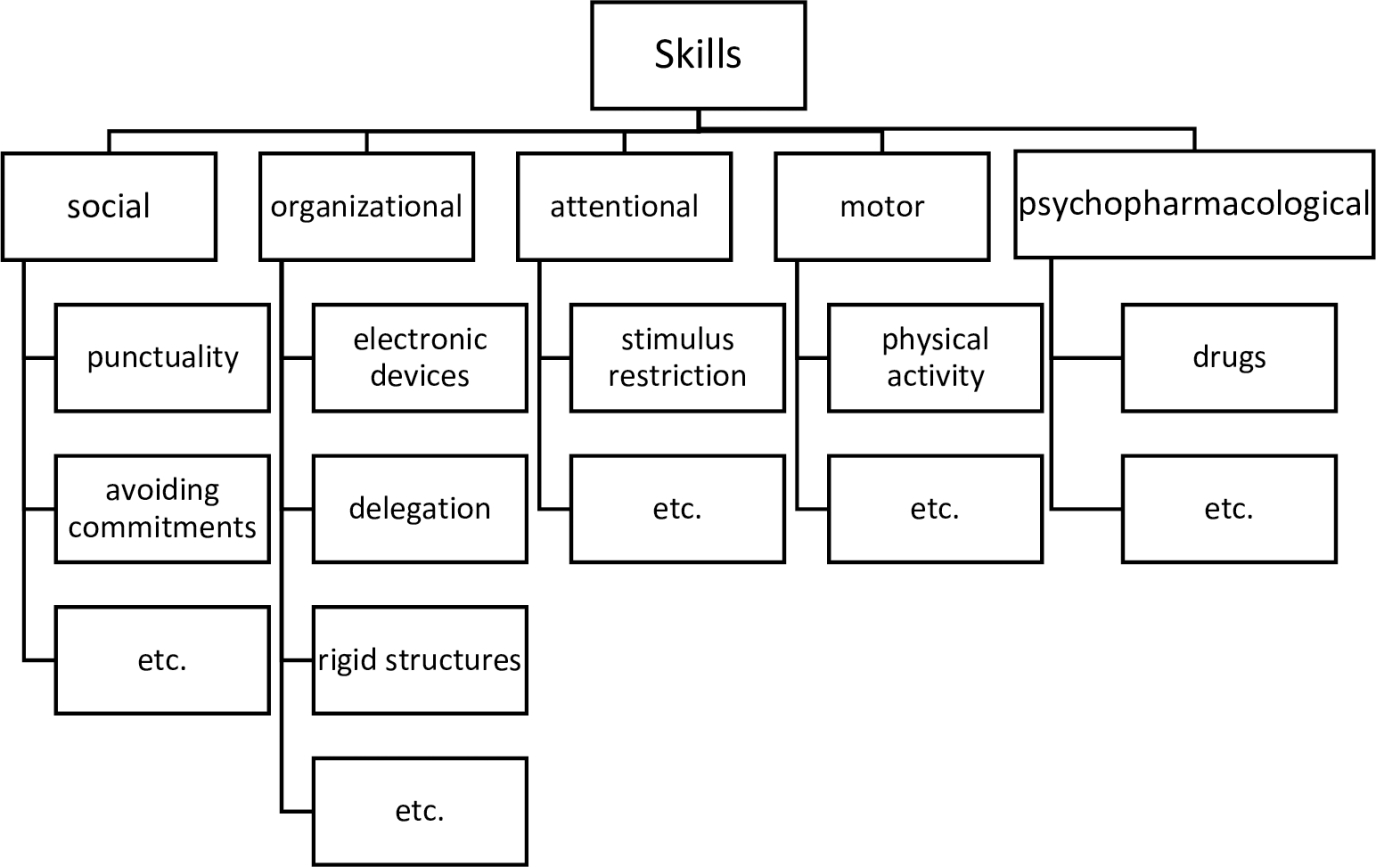
Categories. Reported skills could be roughly organized into five categories: motoric, organizational, social, pharmacological and attentional.

Reported skills could be roughly organized into five categories: motoric, organizational, social, pharmacological and attentional (Fig 1). Interestingly, many patients also perceived some typical ADHD symptoms as their personal skills or strengths, at least partially due to the fact, that instead of controlling or compensating a symptom, they had been able to find or influence an environment in such a way, that this symptom could be perceived as positive. Therefore, we decided to include these ‘skills’ that constitute ADHD-behaviours rather than compensation strategies per se.

### Organizational skills

Inattention and impulsivity both contribute to organizational problems in ADHD patients. It follows then that the most frequently reported skills developed before entering treatment were of an organizational nature. Many patients had learned to use different ‘tricks’ and relied on external devices to improve organization. These skills were used for both activities of daily living and more often in work situations.

Participants reported using checklists and electronic devices to plan or remind them of tasks and appointments.

…because over the years, I have developed strategies to cope with day-to-day work, like really closely structured check lists for every work step. So that I don’t forget anything. Because otherwise, even after three years in this job, I would still forget the correct procedures… VP11

…No, just a reminder app. And in this app there is a number showing the tasks left over. Then I can see, that I have still got five things to do… VP11

…My blackberry is my greatest treasure… VP13

..I write down a lot of things and use a lot of electronic devices. Mobile phone, laptop, tablet, everything but notes, because I lose them. I always end up losing notes or I can’t find them, it’s difficult, that doesn’t work (laughs)… VP20

Depending on the position at work, planning and organizing could also be delegated.

… I have someone, who takes care of making appointments. A secretary… VP3

Several participants reported using special procedures to better cope with impulsivity, aversion to details and attentional problems.

… First, I only work with workmen that work on call, without paper. Everything without paper. With people who think like me, decide like me… VP8

… No paper… I wouldn’t ever even write a letter to my lawyer to give the instructions, ever… VP8

Several participants dealt with attentional problems and restlessness by changing tasks. They claimed this helped them to maintain attention and productivity.

…So if I can’t keep at what I am doing, then I don’t just stare into space or do something unnecessary. I do something else that I had planned on doing, so I can keep a high productivity… VP13

This changing of tasks was perceived as enjoyable. Apparently, such working arrangements were not always consciously sought out due to an awareness of symptoms.

… That is probably why I stayed for so many years at the laboratory…I liked it there. I always had interesting tasks but they never took longer than half an hour. And then I could switch to another device and walk around and there was always something going on… VP12

Two participants (VP8, VP21) reported working independently, which enabled them to plan in such a way, that they could maximize productivity and avoid problems due to loss of attention.

Very rigid structures and detailed plans were seen as helpful or even indispensable by some participants. Particularly the former were perceived to be helpful in attaining higher levels of productivity.

…My week is well planned. I know exactly, what is supposed to happen this week… VP6

…If I compare my daily output with that of a normal person, then I achieve about 150%, just because of what I can compress into a day. And it’s clearly because I am totally structured… VP9

… A rigid daily structure? Yes, even on weekends. I always go to bed at 10, that’s very important, because if I don’t, if I lose this, I start drifting. I get my confused days and I can’t concentrate anymore and I am never happy…of course I would like to sleep in sometimes, but I know, that it won’t be a good day then…VP9

Such rigid structures were reported to be the result of a special educational effort by their parents. It might be surmised, that the parents had recognized the distinct difficulties of their children and adapted accordingly, maybe even without being conscious of the disorder per se.

… Since I was little, it has been ingrained into me to have a structured daily routine. I don’t know, whether it’s because of that that I can perform on such a level…VP9

Thorough planning considering every eventuality also helped reduce discomfort and dysfunctional impulsive reactions.

… I have structured my daily routine myself and have developed strategies for a clear structure. I also always have a plan A, B, C and D. I just couldn’t cope with situations, that weren’t as I had expected, I used to flip. So to avoid this, I started thinking for example when I took the streetcar about when I had to exit and what to do, if I missed it…I just started thinking about all possible eventualities, so that I wouldn’t come up into a situation where I didn’t know what to do… VP6

### Motoric skills

Hyperactivity is another characteristic feature of ADHD, often expressed in adults as restlessness and fidgeting. Physical activity has been said to attenuate some ADHD symptoms [56]. Therefore it is not surprising, that several participants found physical activity to be particularly helpful prior to entering treatment.

… For instance, with sports too, I knew that it helped somewhat… VP7

… I am more productive…As long as I have enough time to do sport. A lot of time for sport… VP9

Even fidgeting was felt to increase productivity and functioning.

… I move my foot the whole time, I’m not even conscious of doing it. But I believe that because of this hyperactivity, I can be very productive, when I concentrate… VP4

Another approach to coping with attentional problems and restlessness was to restrict movement–sometimes even through physical restraints.

… you tie yourself up (in a chair), so that you can’t stand up the whole time. like with a belt, a karate belt, so that you have untie yourself to stand up… it helps you remember, why you were sitting down in the first place… VP21

### Attentional skills

Many participants had also developed strategies to manage distracting stimuli and maintain their attention. Several claimed to work better in environments with fewer stimuli, be it at night or in a cellar.

… it begins when you understand, that there are fewer external stimuli at night, that you are not hungry and that other people are not doing anything…nothing happens, even on the internet, articles are actualized between 12 pm and 7 am… so I just worked at nights…studied at night… VP21

… I used to study in the basement, on a table, it was cold and there wasn’t anything to distract me… VP14

Similar approaches could also be found in social situations, where being in contact with several people at the same time could be perceived as stressful. One participant reported skillfully avoiding scheduling appointments with more than one person, so she could better focus her attention on one person and hide her attentional problems from her environment.

… “Did you never notice, that I only made appointments with one person?”… and if there was an invitation for a bigger party, I always had an excuse, something plausible… VP12

Solitude and environments with fewer uncontrollable stimuli were perceived as helpful in relaxing and were actively sought out.

…nature in general. Just going for a walk or go to the lake or reading. Or, ehm, also cleaning up my place. I like cleaning up. It’s kind of satisfying, a little like cleaning up your thinking. It kind of helps to relax… VP7

… and of course, to be able to cope well with being alone and to even need it at times… VP12

### Social skills

Patients with ADHD often experience interpersonal difficulties due to inattention in conversations, fidgeting, forgetfulness or impulsive behaviour, resulting in blurting out opinions or interrupting conversations. Though the level of impairment in social situations and the level of perception of such problems remained unclear in the interviews, several participants reported the use of special strategies when dealing with others before initiation of therapy.

One of the reported strategies was putting special care into being punctual.

… My compensation strategy is to be overly punctual… VP15

.. and such structures as always being early for an appointment. I am never late… VP6

Since forgetfulness due to inattention or impulsive behaviour may be especially vexing for the environment, another strategy was to consciously avoid rigid commitments requiring special care:

…what I always intuitively did, from early on, was to avoid any kind of dependence/commitment. That became a core principle, to never create any dependence because this brings about commitments. And these restrict me which leads in turn to conflicts… any kind of commitment, even a club membership, where you need to attend meetings five times a year, whatever, even that would have felt restricting…VP8

Behavioural disinhibition was reported to have negative effects in social situations. These could be mitigated by socializing with a high number of friends and colleagues at the same time.

…so by surrounding myself always with lots of people, it never attracted attention when I behaved specially obnoxiously. I always had a reputation for being elusive, for doing my own thing, for not being easily categorizable… I always knew, if I started to have contact like with only five people, in a narrow social environment, there would be trouble. Firstly, people would see through me. And secondly, it would be too restricting for me… VP8

As expected, several participants reported themselves to be especially interesting, funny or entertaining for others–probably due to hyperactivity and impulsiveness. This might be seen as maladaptive behaviour. Alternatively, since it was several times reported as a positive trait, it might also represent an adaptive strategy that does not diminish social functioning per se, but allows the individual to better cope with high levels of restlessness and impatience. Also, being the center of attention in a group might negate problems of inattention in conversations.

… I don’t need to drink, to be so funny or act drunkenly like those who really are. I can do so just as well without being drunk. And I can do it just like that, at once…VP1

…They said, that I could entertain them the whole night, there was always something going on, I had always a lot of energy and was always ready to do something with them and I always had interesting things to tell… VP7

### Psychopharmacological strategies

Many participants reported having had experiences with psychotropic substances. The substances used were cocaine, THC, nicotine, MDMA, amphetamines, alcohol, caffeine, LSD, ‘magic mushrooms’ and benzodiazepines (see Table 2). Some of these substances were perceived to have a beneficial effect on ADHD symptoms and were regularly used for this purpose.

**Table 2:** Drugs.

None		3	9.38%
Legal only		6	18.75%
Illegal		23	71.88%
Substance used			
	Alcohol	21	65.63%
	Nicotine	23	71.88%
	Caffeine	4	12.5%
	Cocaine	11	34.38%
	Cannabis	18	56.25%
	Amphetamines	7	21.88%
	Heroin	2	6.25%
	Hallucinogens	2	6.25%
	Crack	1	3.13%
	Methylphenidat	1	3.13%

Caffeine and alcohol were perceived as having a calming effect.

…I used to drink three to five coffees a day. Nowadays I drink only to enjoy, but then it was purely functional, to calm myself… VP2

…Without Ritalin (Methyphenidate) it was like that: I was standing here and there where like a lot of railways coming towards me and each one had a train on it driving towards me. There were so many impressions, so many requirements or ideas, I felt overwhelmed and I turned around and did nothing. Mainly I used to drink alcohol then, to calm these feelings… VP19

Cocaine was also reported to have a calming effect, to enable sleeping and to enhance concentration.

… Cocaine calmed me down extraordinarily… where others got started, I was just happy to find peace from myself… VP4

… What worked, was that I slept better (with cocaine)… Interestingly, I noticed that I was calmer and could sleep better, it just was more pleasant, in a way… VP8

… During times of partying I noticed, that there was another effect of cocaine, I liked it, but I couldn’t say what it was exactly. And I started taking it regularly, not only at parties. And then, when I was 17 and in my apprenticeship we had an exam and I didn’t even know about what and I hadn’t studied for it. I went in and got a top score, even though I didn’t know beforehand, what this test was about. And I tried it again with cocaine and it worked again and I told myself, that I had to stop… VP11

MDMA was considered to enhance cognitive performance.

…My thoughts were totally clear, I could have achieved a top score in an exam. I was so clear in my head. And I could sleep. I could take greater amounts, had to take greater amounts, to make it work… VP6

THC helped to relax, facilitate sleep and enhance concentration in some cases.

…Because THC is also something, that helps me relax. And helps me sleep, of course…VP7

…What I always continued doing, was smoke pot, and I still do so every evening. To sleep, so I can sleep…VP11

…And I took all my exams while I was stoned. And yes, I could concentrate better or at least I thought so. And I did a lot of work like that, because I felt that it was easier this way… VP12

Nicotine helped in sustaining attention and had a calming effect.

…I need so much kick from nicotine, to focus somewhat. And it doesn’t work for long. That means only for about a minute and then I need to go again…VP15

… How do you treat inner unrest? By smoking cigarettes. It’s like a tingling sensation inside of you. And when I feel it, I go smoke a cigarette. I need to distract myself so I don’t concentrate on that…VP11

Amphetamines were perceived as having a beneficial effect on motivation and prioritizing.

… And then I tried out speed, and it was just what I needed. I was more motivated and the whole way to get there just seemed like no big deal, and you recognized, I’ve got to do this and then this and then I will have reached my goal and have good grades…VP21

## Discussion

To the authors‘ knowledge, this is the first study focusing on skills developed by adult patients with ADHD prior to their diagnosis and their perceptions of these compensatory strategies. Reported skills could be classified into five categories (organizational, motor, social, attentional and psychopharmacological).

The first skill group–organizational skills–was reported most often and reflects the common problems of ADHD patients in organization. Reported strategies highlight the benefits of using modern technology, i.e. smartphones, electronic calendars, reminder apps, etc. and synchronizing them. The developing of particular routines to accommodate typical ADHD behaviours was also seen as helpful. Additionally, the option to change tasks when attention started to diminish was seen as beneficial towards maintaining productivity. Creating special work arrangements, such as using as little paper as possible–because paper can easily be lost–or simplifying hiring and contracting procedures to the point of just having to make one phone call, minimized the risk for procrastination and was seen as well worth the effort.

Tellingly, some participants stressed the need for maintaining rigid structures and claimed that this allowed them to function more efficiently. In this way they could also avoid boredom and mood shifts.

The group of motor skills comprised mainly the use of physical activity. It was perceived as beneficial and for some indispensable in maintaining attention and productivity. A contrasting approach was to restrict movement by binding oneself to the chair to remind the participant of the task at hand when his attention shifted.

The third category of skills included strategies to manage stimuli and attentional problems. Participants reported seeking out environments with relatively few stimuli (i.e. cellar) or confining themselves to working at night. One participant used a similar approach in social life and avoided meeting several people at the same time. Relatively stimulus-poor environments were not only sought out to maximize productivity, but also for their calming and relaxing effect.

The fourth category was comprised of adaptive social skills. These varied greatly and included taking care to be punctual, avoiding commitments demanding too much engagement from the participant or frequent contact with the same people to diminish the impact of social functional impairment on relationships. Since several participants saw themselves as funny, interesting or entertaining and self-reported these characteristics as positive, we decided to include these traits. For some individuals it may represent the best available way to cope with impulsiveness, restlessness and attention problems in social interaction. At the same time, being the focus of attention may have a positive effect on self-esteem, a common problem in ADHD patients [57]. Of special interest is the finding, that several participants saw themselves as being good at manipulating others or sensing the feelings and wishes of others. Whether this constituted a genuine skill or rather a misinterpretation of their own abilities and whether this skill was linked to ADHD or not remained unclear.

The last category comprised the use of psychotropic substances to ameliorate ADHD symptoms. Whereas most patients reported experiences with substance use, only some spontaneously related their substance use with attempts to reduce ADHD symptoms and reported positive effects of caffeine, nicotine, cocaine, MDMA, alcohol, THC and amphetamines on their symptoms.

In our sample we found that many participants were conscious of having used particular strategies to cope with ADHD symptoms prior to diagnosis and treatment. A wide range of coping skills of varying degrees of effectiveness support the assumption, that patients with undiagnosed ADHD spontaneously generate skills they implement regularly.

Although there is a dearth of research on coping mechanisms of patients with ADHD, the main skill groups are consistent with the current state of knowledge, even if some may seem more exotic. Many of the reported organizational skills are reminiscent of psychotherapy programs, as is the management of external stimuli [19, 58, 59]. Mentioned motor skills are also in line with the literature about the benefit of physical activity on ADHD symptoms [56]. The finding that many participants used a variety of psychotropic substances to deliberately reduce ADHD symptoms is also consistent with findings from previous studies [48, 49] and underscores self-medication aspects of co-morbid substance use in ADHD populations. Considering the social impairment associated with ADHD [28, 29, 60] it is unsurprising, that so many patients develop their own strategies to better cope with others in social situations.

Whereas some compensation strategies clearly aimed at controlling symptoms or indirectly compensating them, many participants also reported seeking out special environments or–broadly speaking–adapting them in such a way, that allowed them to function well despite their symptoms. Changing the environment to suit the needs of patients is also consistent with rehabilitation recommendations found in therapy manuals [59]. Interestingly, not all participants were self-aware that the special work situations in which they felt or functioned best, were simultaneously helpful in minimizing loss of functioning due to ADHD symptoms.

### Limitations

Considering that many ADHD symptoms are ego-syntonic and participants in our study had not been prepared for the topics of the interview, it can be assumed they did not report all their coping strategies, as they may not have even been aware of the extent of their adaptation. It may be, that on a repeat interview or with due preparation for the topic some patients may recount more adaptive mechanisms.

Furthermore, our findings only include concrete coping skills. More general abilities of problem solving, stress management, assessment of problems or help-seeking behaviour as reported in a previous study about coping strategies by Young et al. [21] were not investigated in this study. Together with such factors as personality or cognitive abilities such mechanisms may be important for coping with the disorder.

## Conclusions

The results of this study provide insight into how coping strategies are perceived by patients with ADHD, which skills they spontaneously generate and which they find helpful. From a systemic and functional perspective they also underline the positive aspects of ADHD symptoms and behaviours in a particular environment. Furthermore, they may also shed light on the mechanisms underlying late and under-diagnosing. Considering some of the reported, well-developed skills (i.e. rigid structures, managing attentional problems with changing tasks), it may be, that some core symptoms may ‘hide’ behind well-developed coping strategies or particular working environments and this decreases self-awareness of symptoms. Such mechanisms might hamper diagnostic efforts. Lastly, these findings about spontaneously developed coping strategies improve insight and understanding of the development of patients with ADHD during late adolescence and adulthood. In turn, this might help further therapeutic alliance and patient collaboration and may even serve as concrete guides for therapeutic interventions.

## References

[1] Hill JC, Schoener EP. Age-dependent decline of attention deficit hyperactivity disorder. Am J Psychiatry. 1996;153(9):1143–6. doi: 10.1176/ajp.153.9.1143 . 8780416 10.1176/ajp.153.9.1143

[2] Rösler M, Casas M, Konofal E, Buitelaar J. Attention deficit hyperactivity disorder in adults. World Journal of Biological Psychiatry. 2010;11(5):684–98. doi: 10.3109/15622975.2010.483249 20521876 10.3109/15622975.2010.483249

[3] Spencer T, Biederman J, Wilens TE, Faraone SV. Adults with attention-deficit/hyperactivity disorder: a controversial diagnosis. The Journal of clinical psychiatry. 1997;59:59–68. 9680054

[4] Faraone SV, Biederman J, Mick E. The age-dependent decline of attention deficit hyperactivity disorder: a meta-analysis of follow-up studies. Psychol Med. 2006;36(2):159–65. doi: 10.1017/S003329170500471X . 16420712 10.1017/S003329170500471X

[5] Faraone SV, Biederman J, Spencer T, Wilens T, Seidman LJ, Mick E, et al Attention-deficit/hyperactivity disorder in adults: an overview. Biological psychiatry. 2000;48(1):9–20. 10913503 10.1016/s0006-3223(00)00889-1

[6] Fayyad J, De Graaf R, Kessler R, Alonso J, Angermeyer M, Demyttenaere K, et al Cross–national prevalence and correlates of adult attention–deficit hyperactivity disorder. The British Journal of Psychiatry. 2007;190(5):402–9. 17470954 10.1192/bjp.bp.106.034389

[7] Simon V, Czobor P, Bálint S, Mészáros Á, Bitter I. Prevalence and correlates of adult attention-deficit hyperactivity disorder: meta-analysis. The British Journal of Psychiatry. 2009;194(3):204–11. doi: 10.1192/bjp.bp.107.048827 19252145 10.1192/bjp.bp.107.048827

[8] Kessler R. The Prevalence and Correlates of Adult ADHD in the United States: Results From the National Comorbidity Survey Replication. American Journal of Psychiatry. 2006;163(4):716 doi: 10.1176/appi.ajp.163.4.716 16585449 10.1176/appi.ajp.163.4.716 PMC2859678

[9] Stevens SE, Sonuga-Barke EJ, Kreppner JM, Beckett C, Castle J, Colvert E, et al Inattention/overactivity following early severe institutional deprivation: presentation and associations in early adolescence. Journal of abnormal child psychology. 2008;36(3):385–98. doi: 10.1007/s10802-007-9185-5 17965931 10.1007/s10802-007-9185-5

[10] Banerjee TD, Middleton F, Faraone SV. Environmental risk factors for attention‐deficit hyperactivity disorder. Acta paediatrica. 2007;96(9):1269–74. doi: 10.1111/j.1651-2227.2007.00430.x 17718779 10.1111/j.1651-2227.2007.00430.x

[11] Faraone SV, Perlis RH, Doyle AE, Smoller JW, Goralnick JJ, Holmgren MA, et al Molecular genetics of attention-deficit/hyperactivity disorder. Biological psychiatry. 2005;57(11):1313–23. doi: 10.1016/j.biopsych.2004.11.024 15950004 10.1016/j.biopsych.2004.11.024

[12] Kuntsi J, Rijsdijk F, Ronald A, Asherson P, Plomin R. Genetic influences on the stability of attention-deficit/hyperactivity disorder symptoms from early to middle childhood. Biological Psychiatry. 2005;57(6):647–54. doi: 10.1016/j.biopsych.2004.12.032 15780852 10.1016/j.biopsych.2004.12.032

[13] Larsson J-O, Larsson H, Lichtenstein P. Genetic and environmental contributions to stability and change of ADHD symptoms between 8 and 13 years of age: a longitudinal twin study. Journal of the American Academy of Child & Adolescent Psychiatry. 2004;43(10):1267–75. 15381894 10.1097/01.chi.0000135622.05219.bf

[14] Bekker E, Overtoom C, Kenemans J, Kooij J, De Noord I, Buitelaar J, et al Stopping and changing in adults with ADHD. Psychological medicine. 2005;35(06):807–16. 15997601 10.1017/s0033291704003459

[15] Seidman LJ, Valera EM, Bush G. Brain function and structure in adults with attention-deficit/hyperactivity disorder. Psychiatric Clinics of North America. 2004;27(2):323–47. doi: 10.1016/j.psc.2004.01.002 15064000 10.1016/j.psc.2004.01.002

[16] Seidman LJ, Valera EM, Makris N. Structural brain imaging of attention-deficit/hyperactivity disorder. Biological psychiatry. 2005;57(11):1263–72. doi: 10.1016/j.biopsych.2004.11.019 15949998 10.1016/j.biopsych.2004.11.019

[17] Marije Boonstra A, Oosterlaan J, Sergeant JA, Buitelaar JK. Executive functioning in adult ADHD: a meta-analytic review. Psychological medicine. 2005;35(08):1097–108. 16116936 10.1017/s003329170500499x

[18] Frodl T, Skokauskas N. Meta‐analysis of structural MRI studies in children and adults with attention deficit hyperactivity disorder indicates treatment effects. Acta Psychiatrica Scandinavica. 2012;125(2):114–26. doi: 10.1111/j.1600-0447.2011.01786.x 22118249 10.1111/j.1600-0447.2011.01786.x

[19] Kooij SJ, Bejerot S, Blackwell A, Caci H, Casas-Brugué M, Carpentier PJ, et al European consensus statement on diagnosis and treatment of adult ADHD: The European Network Adult ADHD. BMC psychiatry. 2010;10(1):67. 20815868 10.1186/1471-244X-10-67 PMC2942810

[20] Wender PH, Wolf LE, Wasserstein J. Adults with ADHD. Annals of the New York Academy of Sciences. 2001;931(1):1–16. 11462736

[21] Young S. Coping strategies used by adults with ADHD. Personality and Individual Differences. 2005;38(4):809–16. doi: 10.1016/j.paid.2004.06.005

[22] Barkley RA, Fischer M, Smallish L, Fletcher K. Young adult outcome of hyperactive children: adaptive functioning in major life activities. Journal of the American Academy of Child & Adolescent Psychiatry. 2006;45(2):192–202. 16429090 10.1097/01.chi.0000189134.97436.e2

[23] Biederman J, Petty CR, Fried R, Kaiser R, Dolan CR, Schoenfeld S, et al Educational and occupational underattainment in adults with attention-deficit/hyperactivity disorder: a controlled study. Journal of Clinical Psychiatry. 2008. 10.4088/jcp.v69n0803 18681752

[24] Halmoy A, Fasmer OB, Gillberg C, Haavik J. Occupational outcome in adult ADHD: impact of symptom profile, comorbid psychiatric problems, and treatment: a cross-sectional study of 414 clinically diagnosed adult ADHD patients. J Atten Disord. 2009;13(2):175–87. doi: 10.1177/1087054708329777 . 19372500 10.1177/1087054708329777

[25] Eakin L, Minde K, Hechtman L, Ochs E, Krane E, Bouffard R, et al The marital and family functioning of adults with ADHD and their spouses. Journal of Attention Disorders. 2004;8(1):1–10. doi: 10.1177/108705470400800101 15669597 10.1177/108705470400800101

[26] Robin AL, Payson E. The impact of ADHD on marriage. The ADHD Report. 2002;10(3):9–14.

[27] Friedman SR, Rapport LJ, Lumley M, Tzelepis A, VanVoorhis A, Stettner L, et al Aspects of social and emotional competence in adult attention-deficit/hyperactivity disorder. Neuropsychology. 2003;17(1):50 12597073

[28] Able SL, Johnston JA, Adler LA, Swindle RW. Functional and psychosocial impairment in adults with undiagnosed ADHD. Psychological medicine. 2007;37(01):97–107. 16938146 10.1017/S0033291706008713

[29] Harpin V, Mazzone L, Raynaud JP, Kahle J, Hodgkins P. Long-Term Outcomes of ADHD: A Systematic Review of Self-Esteem and Social Function. J Atten Disord. 2016;20(4):295–305. doi: 10.1177/1087054713486516 . 23698916 10.1177/1087054713486516

[30] Barkley RA, Murphy KR, Dupaul GJ, Bush T. Driving in young adults with attention deficit hyperactivity disorder: Knowledge, performance, adverse outcomes, and the role of executive functioning. Journal of the International Neuropsychological Society. 2002;8(05):655–72. 12164675 10.1017/s1355617702801345

[31] Fischer M, Barkley RA, Smallish L, Fletcher K. Hyperactive children as young adults: Driving abilities, safe driving behavior, and adverse driving outcomes. Accident Analysis & Prevention. 2007;39(1):94–105. 16919226 10.1016/j.aap.2006.06.008

[32] Frei A, Hornung R, Eich D. [Tobacco consumption of adults diagnosed with ADHD]. Nervenarzt. 2010;81(7):860–6. doi: 10.1007/s00115-009-2922-y . 20111852 10.1007/s00115-009-2922-y

[33] Ohlmeier MD, Peters K, Te Wildt BT, Zedler M, Ziegenbein M, Wiese B, et al Comorbidity of alcohol and substance dependence with attention-deficit/hyperactivity disorder (ADHD). Alcohol and alcoholism. 2008;43(3):300–4. doi: 10.1093/alcalc/agn014 18326548 10.1093/alcalc/agn014

[34] McGough JJ, Smalley SL, McCracken JT, Yang M, Del'Homme M, Lynn DE, et al Psychiatric comorbidity in adult attention deficit hyperactivity disorder: findings from multiplex families. American Journal of Psychiatry. 2005;162(9):1621–7. doi: 10.1176/appi.ajp.162.9.1621 16135620 10.1176/appi.ajp.162.9.1621

[35] Secnik K, Swensen A, Lage MJ. Comorbidities and costs of adult patients diagnosed with attention-deficit hyperactivity disorder. Pharmacoeconomics. 2005;23(1):93–102. 15693731 10.2165/00019053-200523010-00008

[36] Mészáros Á, Czobor P, Bálint S, Komlósi S, Simon V, Bitter I. Pharmacotherapy of adult attention deficit hyperactivity disorder (ADHD): a meta-analysis. International journal of neuropsychopharmacology. 2009;12(8):1137–47. doi: 10.1017/S1461145709990198 19580697 10.1017/S1461145709990198

[37] Faraone SV, Spencer T, Aleardi M, Pagano C, Biederman J. Meta-analysis of the efficacy of methylphenidate for treating adult attention-deficit/hyperactivity disorder. Journal of clinical psychopharmacology. 2004;24(1):24–9. doi: 10.1097/01.jcp.0000108984.11879.95 14709943 10.1097/01.jcp.0000108984.11879.95

[38] Excellence NIfC. Attention deficit hyperactivity disorder: the NICE guideline on diagnosis and management of ADHD in children, young people and adults The British Psychological Society and the Royal College of Psychiatrists, London 2009. 22420012

[39] Young S, Amarasinghe JM. Practitioner review: Non-pharmacological treatments for ADHD: a lifespan approach. J Child Psychol Psychiatry. 2010;51(2):116–33. doi: 10.1111/j.1469-7610.2009.02191.x . 19891745 10.1111/j.1469-7610.2009.02191.x

[40] Safren SA, Otto MW, Sprich S, Winett CL, Wilens TE, Biederman J. Cognitive-behavioral therapy for ADHD in medication-treated adults with continued symptoms. Behav Res Ther. 2005;43(7):831–42. doi: 10.1016/j.brat.2004.07.001 . 15896281 10.1016/j.brat.2004.07.001

[41] Solanto MV, Marks DJ, Wasserstein J, Mitchell K, Abikoff H, Alvir JMJ, et al Efficacy of meta-cognitive therapy for adult ADHD. 2014. 10.1176/appi.ajp.2009.09081123 PMC3633586 20231319

[42] Bramham J, Young S, Bickerdike A, Spain D, McCartan D, Xenitidis K. Evaluation of group cognitive behavioral therapy for adults with ADHD. Journal of attention disorders. 2008. 10.1177/1087054708314596 18310557

[43] Ramsay JR. CBT for Adult ADHD: Adaptations and Hypothesized Mechanisms of Change. Journal of Cognitive Psychotherapy. 2010;24(1):37–45. doi: 10.1891/0889-8391.24.1.37

[44] Asherson P, Akehurst R, Kooij JJ, Huss M, Beusterien K, Sasane R, et al Under diagnosis of adult ADHD: cultural influences and societal burden. J Atten Disord. 2012;16(5 Suppl):20S–38S. doi: 10.1177/1087054711435360 . 22377849 10.1177/1087054711435360

[45] Barkley RA, Anastopoulos AD, Guevremont DC, Fletcher KE. Adolescents with ADHD: patterns of behavioral adjustment, academic functioning, and treatment utilization. Journal of the American Academy of Child & Adolescent Psychiatry. 1991;30(5):752–61. 1938790 10.1016/s0890-8567(10)80010-3

[46] Levin ED, Conners C, Sparrow E, Hinton SC, Erhardt D, Meck W, et al Nicotine effects on adults with attention-deficit/hyperactivity disorder. Psychopharmacology. 1996;123(1):55–63. doi: 10.1007/BF02246281 8741955 10.1007/BF02246281

[47] Biederman J, Wilens T, Mick E, Milberger S, Spencer TJ, Faraone SV. Psychoactive substance use disorders in adults with attention deficit hyperactivity disorder (ADHD): effects of ADHD and psychiatric comorbidity. American Journal of Psychiatry. 1995;152(11):1652–8. doi: 10.1176/ajp.152.11.1652 7485630 10.1176/ajp.152.11.1652

[48] Liebrenz M, Frei A, Fisher CE, Gamma A, Buadze A, Eich D. Adult attention-deficit/hyperactivity disorder and nicotine use: a qualitative study of patient perceptions. BMC Psychiatry. 2014;14:141 doi: 10.1186/1471-244X-14-141 ; PubMed Central PMCID: PMCPMC4037284. 24885526 10.1186/1471-244X-14-141 PMC4037284

[49] Nehlin C, Nyberg F, Oster C. The patient's perspective on the link between ADHD and substance use: a qualitative interview study. J Atten Disord. 2015;19(4):343–50. doi: 10.1177/1087054714554618 . 25359762 10.1177/1087054714554618

[50] Organization WH. The ICD-10 classification of mental and behavioural disorders: clinical descriptions and diagnostic guidelines: Geneva: World Health Organization; 1992.

[51] Dicicco-Bloom B, Crabtree BF. The qualitative research interview. Med Educ. 2006;40(4):314–21. doi: 10.1111/j.1365-2929.2006.02418.x . 16573666 10.1111/j.1365-2929.2006.02418.x

[52] Britten N. Qualitative interviews in medical research. BMJ. 1995;311(6999):251–3. ; PubMed Central PMCID: PMCPMC2550292. 7627048 10.1136/bmj.311.6999.251 PMC2550292

[53] Gill P, Stewart K, Treasure E, Chadwick B. Methods of data collection in qualitative research: interviews and focus groups. Br Dent J. 2008;204(6):291–5. doi: 10.1038/bdj.2008.192 . 18356873 10.1038/bdj.2008.192

[54] Mayring P, editor Combination and integration of qualitative and quantitative analysis. Forum Qualitative Sozialforschung/Forum: Qualitative Social Research; 2001.

[55] Tong A, Sainsbury P, Craig J. Consolidated criteria for reporting qualitative research (COREQ): a 32-item checklist for interviews and focus groups. Int J Qual Health Care. 2007;19(6):349–57. doi: 10.1093/intqhc/mzm042 . 17872937 10.1093/intqhc/mzm042

[56] Gapin JI, Labban JD, Etnier JL. The effects of physical activity on attention deficit hyperactivity disorder symptoms: the evidence. Prev Med. 2011;52 Suppl 1:S70–4. doi: 10.1016/j.ypmed.2011.01.022 . 21281664 10.1016/j.ypmed.2011.01.022

[57] Shaw-Zirt B, Popali-Lehane L, Chaplin W, Bergman A. Adjustment, social skills, and self-esteem in college students with symptoms of ADHD. J Atten Disord. 2005;8(3):109–20. doi: 10.1177/1087054705277775 . 16009659 10.1177/1087054705277775

[58] Kooij JJS. Adult ADHD—Diagnostic Assessment and Treatment. Third Edition ed. London: Springer; 2013.

[59] Ramsay JRR, Anthony L. Cognitive-Behavioral Therapy for Adult ADHD: An Integrative Psychosocial and Medical Approach. 2 ed: Routledge; 2014 254 p.

[60] Harpin VA. The effect of ADHD on the life of an individual, their family, and community from preschool to adult life. Archives of disease in childhood. 2005;90(suppl 1):i2–i7. 15665153 10.1136/adc.2004.059006 PMC1765272

